# Recent Advances in the Biological Investigation of Organometallic Platinum-Group Metal (Ir, Ru, Rh, Os, Pd, Pt) Complexes as Antimalarial Agents

**DOI:** 10.3390/molecules25225276

**Published:** 2020-11-12

**Authors:** Mziyanda Mbaba, Taryn M. Golding, Gregory S. Smith

**Affiliations:** Department of Chemistry, Faculty of Science, University of Cape Town, Rondebosch, Cape Town 7700, South Africa; mziyanda.mbaba@uct.ac.za (M.M.); GLDTAR006@myuct.ac.za (T.M.G.)

**Keywords:** bioorganometallic chemistry, malaria, *Plasmodium falciparum*, platinum-group metals, mechanism of action

## Abstract

In the face of the recent pandemic and emergence of infectious diseases of viral origin, research on parasitic diseases such as malaria continues to remain critical and innovative methods are required to target the rising widespread resistance that renders conventional therapies unusable. The prolific use of auxiliary metallo-fragments has augmented the search for novel drug regimens in an attempt to combat rising resistance. The development of organometallic compounds (those containing metal-carbon bonds) as antimalarial drugs has been exemplified by the clinical development of ferroquine in the nascent field of Bioorganometallic Chemistry. With their inherent physicochemical properties, organometallic complexes can modulate the discipline of chemical biology by proffering different modes of action and targeting various enzymes. With the beneficiation of platinum group metals (PGMs) in mind, this review aims to describe recent studies on the antimalarial activity of PGM-based organometallic complexes. This review does not provide an exhaustive coverage of the literature but focusses on recent advances of bioorganometallic antimalarial drug leads, including a brief mention of recent trends comprising interactions with biomolecules such as heme and intracellular catalysis. This resource can be used in parallel with complementary reviews on metal-based complexes tested against malaria.

## 1. Introduction

The turn of the 21st century has seen great developments and efforts directed toward containing and eradicating the spread of infectious diseases around the globe. Drug research and innovation within the pharmaceutical industry and academia, as well as government vaccination campaigns, are at the forefront of these endeavours. The elimination of wildtype cases of the infectious viral disease, polio, which has been recently declared “eradicated” from the African continent, attests to the progress made within the field of infectious diseases [1]. Despite these successes, infectious diseases like malaria remain prevalent and still burden the health-care sector and threaten the lives of many in endemic regions in Sub-Saharan Africa and parts of Asia. Of the estimated 228 million cases of malaria, in 2018, Africa was reported to have borne the largest burden, accounting for approximately 93% (213 million) of these cases [2]. Unfortunately, treatment of this disease is proving challenging, due in part to the emergence of new, tenacious strains of the malaria parasite that are displaying resistance to current frontline drugs used to treat this disease. Of the five *Plasmodium* species known to cause malaria, *Plasmodium falciparum* is the most virulent, and according to the World Health Organisation accounted for more than 400,000 deaths in 2018 alone [2]. The plight of antimalarial drug resistance prompted the introduction of artemisinin-based combination therapies (ACTs), which have proven fruitful in this struggle, as this treatment regimen involves combining antimalarials with independent modes of action to improve treatment outcomes and counter the threat of resistance [3,4]. However, reports of *Plasmodium* strains developing resistance to ACTs have already been recorded [5]. This demands an expansion of the antimalarial drug arsenal and search for innovative drug candidates possessing novel mechanistic modalities unknown to the malaria parasite to combat the development of clinical resistance.

The application of bioactive metallic compounds has been extensively explored, in contemporary drug discovery, as nonconventional therapeutic chemotypes possessing novel, and sometimes unique modes of action, compared with their traditional organic counterparts [6]. The success of metallodrugs was accentuated by the seminal discovery of the platinum-based anticancer agent, cisplatin, which has had tremendous success within cancer treatment regimens [7,8]. Concerning its pharmacological applications, platinum is the archetype for a group of related metals, known as platinum-group metals (PGMs), i.e., iridium, ruthenium, rhodium, osmium, platinum, and palladium, which share many similarities in both their physical and chemical attributes [9]. The ability of PGMs to form multiple coordination bonds with electron-rich atoms, such as nitrogen, oxygen, and sulfur, from various ligands, allows for the incorporation of these metallic centres into known chemical scaffolds. Consequently, PGM complexes are a diverse group of compounds, which can be easily fine-tuned, resulting in widespread potential applications. These complexes have been the subject of much research interest within fields of catalysis and biomedical applications, particularly cancer, having reported many success stories [10,11]. Their biomedical applications, in particular, have been exhaustively covered in the literature, with recent reviews comprehensively summarizing the biological activity of PGMs, including their anticancer, antibacterial, and antiviral properties [12,13,14,15]. Selected PGM compounds, like ruthenium complexes, are showing great promise as light-activated cytotoxic agents, in the emerging field of photodynamic therapy, for cancer treatment [16,17].

Likewise, the introduction of PGM moieties into known pharmacological scaffolds, possessing antiplasmodial activity, has been shown to enhance the biological activity of these organic compounds. This approach offers an opportunity to restore the activity of antimalarial drugs, already suffering full-blown clinical resistance, while displaying increased activity toward resistant *P. falciparum* strains [18,19,20]. Most reviews covering the biological activity of PGM complexes, including malaria, have predominantly focused on inorganic complexes, i.e., compounds with coordinate bonds between the metal and a heteroatom. In this reference work, we highlight the antimalarial activity of organometallic PGM complexes comprising at least one metal-carbon covalent bond, as well as their plausible/proposed mechanisms of action, which have been documented in the literature. With respect to current trends in research activities on PGM complexes, we have emphasized recent developments within this field published over the past five years.

## 2. Iridium Complexes

**Figure 1:**
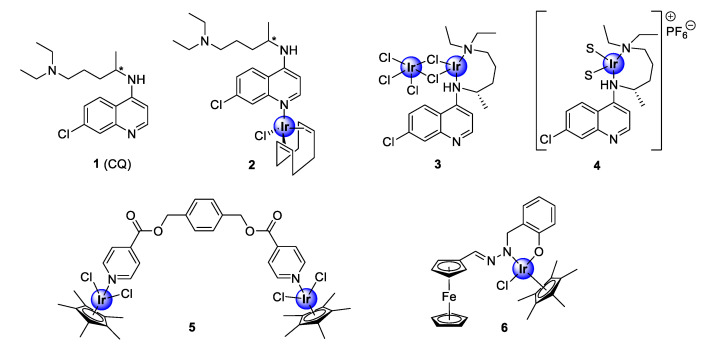
Chemical structures of chloroquine (**1**), the first antimalarial iridium-chloroquine complexes (**2**–**4**), and early examples of non-quinoline organoiridium complexes (**5**–**6**) [21,22,23].

Iridium complexes are among the most studied complexes for their biological activity, within the PGM family of coordination compounds. Organometallic iridium compounds predominantly consist of half-sandwich pentamethylcyclopentadienyl-coordinated chemical entities that adopt the well-known three-legged piano-stool conformation. The investigation of iridium-based compounds, as antimalarial agents, was brought into prominence by the seminal study of Navarro and colleagues in 2009, who incorporated iridium into the scaffold of the quinolinyl clinical antimalarial drug, chloroquine (**1**, CQ), to generate new antiplasmodial iridium complexes (**2**–**4**) [21]. These complexes displayed efficacy comparable to the parental drug against a chemosensitive *P. berghei* malarial strain (Figure 1) [21]. Most notably, complex **2** demonstrated superior activity to chloroquine. Since then, iridium complexes of non-chloroquine-based scaffolds, such as pyridyl esters (**5**) and salicylaldimine (**6**), have been expansively studied for their inhibitory effects against both drug-resistant and -sensitive strains of the *Plasmodium* parasite (Figure 1) [22,23].

The incorporation of the pentamethylcyclopentadienyl (Cp*) ligand is favoured over the unsubstituted cyclopentadienyl (Cp) congener, owing to its increased lipophilicity and higher stability [24]. This enhanced stability is ascribed to the combined effects of steric shielding of the metallic centre and the greater electron density within the Cp* ligand [24]. For chloroquine-derived organometallic iridium complexes, the enhanced lipophilicity of the Cp* ligand is hypothesized to confer an overall improvement in the lipophilicity of the resultant compound, leading to increased retention within the active site of the parasite, i.e., digestive vacuole (DV), making this entity attractive to thwart the development of drug resistance [24].

### 2.1. Quinoline-Salicylaldiminato/Picolamine/Imidazole Ligands

**Figure 2:**
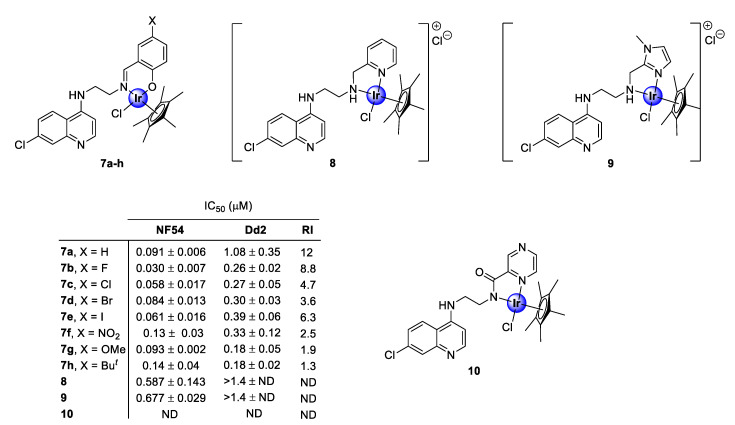
Chemical structures of *N^N*- and *N^O*-coordinated IrCp* complexes and their corresponding IC_50_ values [24,25].

In 2016, Nordlander and co-workers investigated the antiplasmodial activity of *N^N*- and *N^O*-coordinated IrCp* complexes, based on chloroquine analogues of salicylaldimine (**7a**–**h**), 2-picolamine (**8**), and 2-aminomethylimidazole (**9**) as ligands (Figure 2) [24]. The uncoordinated ligands, as well as the corresponding complexes, were screened for their antiplasmodial activity against the chloroquine-sensitive (CQS) NF54 strain and the chloroquine -resistant (CQR) Dd2 strain of the malaria parasite, *P. falciparum*. Generally, the complexes exhibited activity in the sub-micromolar range (0.030–0.677 μM), albeit lower than that of the corresponding ligands, with higher selectivity for the NF54 CQS strain. However, the majority of the tested complexes interestingly showed lower resistant indices (RI) compared with the ligands, which is a good indication of reduced cross-resistance upon complexation, with compounds **7e** and **7g**–**h** displaying increased efficacy against the chloroquine-resistant Dd2 (CQR) strain, compared with their corresponding ligands. Elucidation of the structure-activity relationship (SAR) analysis of the salicylaldiminato IrCp* complexes (**7a**–**h**) revealed no clear trend for the CQR strain, however, a linear correlation depicting an increase in activity upon substitution with electron-withdrawing groups (EWGs) was observed for the CQS strain. It should also be noted that the salicylaldimine ligand was more favorable for plasmodial potency, relative to the picolamine and imidazole ligands. A subsequent study, reported in the following year by the same research group, revealed that coordination with a pyrazine amide ligand (**10**) was detrimental for antiplasmodial activity, as the resulting complex was inactive at the highest tested concentration against the NF54 parasite strain (Figure 2) [25].

**Figure 3:**
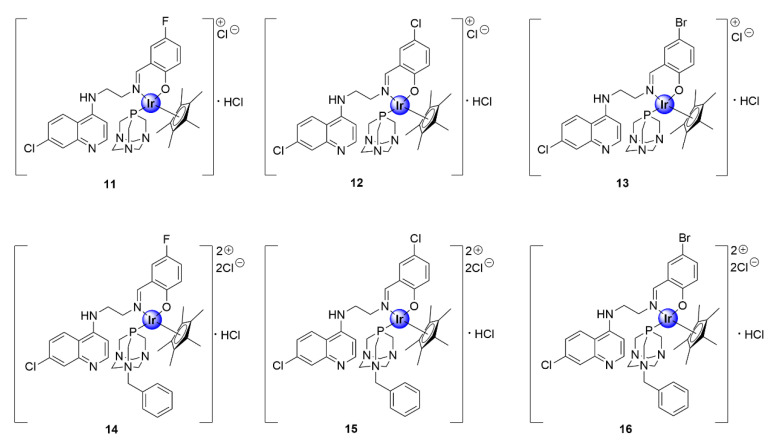
1,3,5-triaza-phosphaadamantane (PTA)-derived IrCp* complexes featuring the chloroquine scaffold [26].

In another study, the Smith and Nordlander groups evaluated the antimalarial activity of IrCp* complexes, based on the chloroquine nucleus, with *N^O^P*-coordination via salicylaldimine and 1,3,5-triaza-phosphaadamantane (PTA) ligands (Figure 3) [26]. The incorporation of the water-soluble PTA ligand was inspired by the medicinal attributes of this moiety, which was observed within ruthenium(II)-arene PTA (RAPTA) complexes, reported by the Dyson group, which showed antiproliferative efficacy against several cancer cell-lines [27]. The multiple basic nitrogen atoms within the PTA unit increases the overall basicity of the resultant complex, providing multiple sites for protonation once in the DV of the parasite, which is a feature crucial for the activity of antimalarial drugs, particularly 4-aminoquinolines [28]. The PTA complexes, isolated as hydrochloride salts, displayed antiplasmodial activity against CQS NF54 and CQR K1 strains of *P. falciparum*, with IC_50_ values ranging between 0.11 and 1.7 μM [26]. The benzyl-PTA congeners (**14**–**16**) were slightly more efficacious than their non-benzylated counterparts (**11**–**13**), against both parasite strains. Furthermore, selected complexes (**11**, **12**, and **15**) were screened for their in vitro cytotoxicity against mammalian Chinese hamster ovarian (CHO) cells. These complexes displayed reduced cytotoxicity toward the CHO cell-line, and thus greater selectivity toward the malaria parasite, with selectivity indices greater than or equal to 10. The increased potency of the benzyl-PTA complexes (**14**–**16**) may be rationalized by their enhanced lipophilicity, owing to the presence of the benzyl unit. Mechanistic studies further suggested that the complexes acted via the inhibition of hemozoin synthesis, which is a pathway unique to the malaria parasite and a crucial detoxification mechanism.

### 2.2. Quinoline-Triazole Ligands

**Figure 4:**
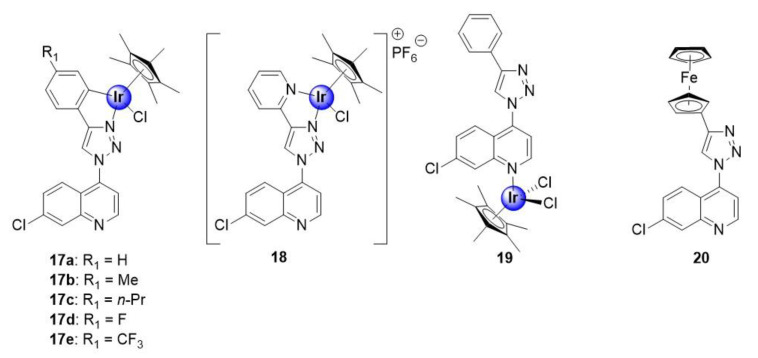
Neutral *C^N*-chelating half-sandwich Ir(III)Cp* complexes and monodentate *N*-coordinated analogues, with in vitro antimalarial activity targeting hemozoin inhibition and the NAD^+^/NADH cycle, by intracellular catalysis [29].

Melis et al. recently reported on a series of quinoline-triazole Ir(III)Cp* conjugates, shown to inhibit the growth of NF54 and K1 strains of *P. falciparum*, with activities ranging between 0.25–2.34 μM and 0.65–3.06 μM, respectively (Figure 4) [29]. Most notably, the activities of the complexes are substantially superior to those of the corresponding uncoordinated ligands, against the NF54 strain, with the activity, in some cases, exceeding that of the ligand by over 20-fold. This result highlights the potential benefits of metal incorporation, within drug design, offering an opportunity to enhance the pharmacological efficacy of organic compounds upon complexation. The cationic *N^N*-coordinated pyridyl complex **18** was the most active in the series, against both the sensitive and resistant strains, with IC_50_ values of 0.25 ± 0.11 and 0.63 ± 0.06 μM, respectively. Furthermore, the selectivity indices for the tested complexes (**17a**–**e**, **18**, and **20**) suggest that they are not cytotoxic toward the CHO-cell line, and thus display increased selectivity for the parasite, with complex **18** almost 500 times more selective toward parasitic cells than the mammalian cell-line. It was also noted that the incorporation of hydrophobic substituents did not seem to influence the antiplasmodial activity of the metal-based compounds. Furthermore, coordination at the quinoline nitrogen atom (**19**) was found to be unfavourable for activity. Finally, preliminary mechanistic studies suggested a dual mode of action by the compounds, involving inhibition of the hemozoin pathway and perturbation of the NAD^+^/NADH biochemical process by intracellular catalysis, which will be discussed in Section 7.

### 2.3. Sulfadoxine Ligands

**Figure 5:**
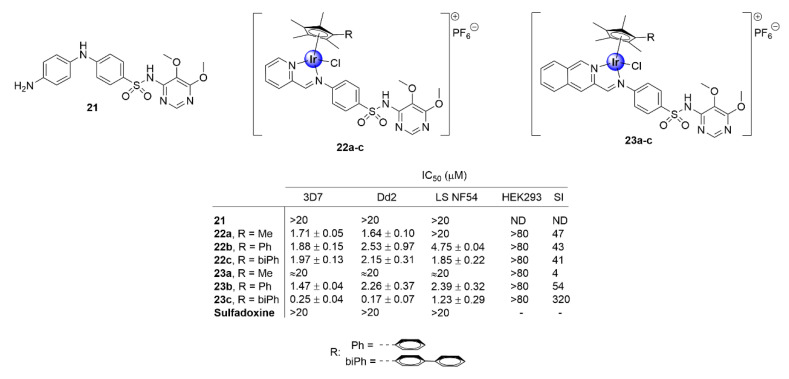
Chemical structures of IrCp* complexes based on the sulfadoxine scaffold and their corresponding IC_50_ values [30].

Apart from the derivatization of chloroquine, appending IrCp* moieties to other known drug scaffolds has proven to be a successful strategy to enhance the biological activity of the parental organic compound, while imparting potentially new and beneficial pharmacological properties. Most notably, complexation with the IrCp* moiety has been shown to restore the potency of antimalarial drugs that have experienced total clinical resistance. Chellan and colleagues demonstrated this upon their exploration of *N^N*-chelated iridium analogues of the clinical antimalarial drug, sulfadoxine (**21**), which is facing full resistance by *P. falciparum* strains (Figure 5) [30]. This group synthesized a series of *N,N′*-chelate pyridylimino- (**22**) or quinolylimino- (**23**) IrCp* complexes, functionalized with sulfadoxine (Figure 5), and evaluated their antiplasmodial activity against *P. falciparum* strains. The complexes inhibited the growth of CQS (3D7 and late stage NF54 gametocytes) and CQR (Dd2) strains of *P. falciparum*, with IC_50_ values in the sub- and low micromolar range, with no significant toxicity towards non-pathogenic human embryonic kidney cells (HEK293). Annulation of the IrCp* moiety with phenyl and biphenyl units was desirable for activity, as shown by the increased efficacy of the complexes endowed with these rings, a fact that could be attributed to increased lipophilicity. This is further corroborated by the superior activity of the bulkier isoquinolyl complexes (**23a**–**c**) compared with their pyridyl congeners (**22a**–**c**). Most significantly, the parental drug sulfadoxine did not show any toxicity toward the investigated *P. falciparum* strains, at the highest tested concentration, thus illustrating the practicality of incorporating an IrCp* moiety into drug scaffolds as a viable approach for restoring the activity of clinical drugs facing resistance.

### 2.4. Benzimidazole (Hybrid) Ligands

**Figure 6:**
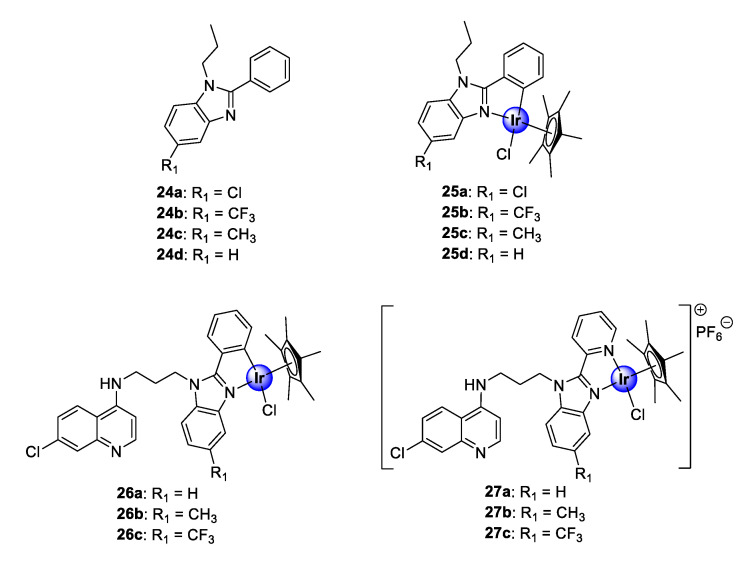
Chemical structures of antimalarial 2-phenylbenzimidazole ligands (**24a**–**d**) and their corresponding IrCp* complexes (**25a**–**d**) as well as neutral and cationic quinolinyl-benzimidazole hybrids (**26**–**27**) [31,32].

Recently, the research group of Smith reported a series of neutral, cyclometallated Ir(III)Cp* complexes (**25**), based on the 2-phenylbenzimidazole scaffold (**24**), showing in vitro antiplasmodial activity against NF54 and K1 strains of *P. falciparum* (Figure 6) [31]. The synthesized complexes displayed activities in the low micromolar range against the tested strains. Most noteworthy was the significant increase in activity observed upon metal complexation of the ligands (**24a**–**d**) with IrCp*, which enhanced the activity of ligands **24a**–**24c** by 10- to 18-fold. Complexation of **24d**, however, resulted in compound **25d** being approximately 117 times more active than the uncoordinated ligand against the CQS NF54 strain, further highlighting the medicinal benefits of incorporating organometallic fragments into organic scaffolds. The complexes (**25a**–**d**), however, were less selective for the CQR K1 strain, as indicated by their lower activity. Evaluation of substituent effects on activity shows that substitution with CF_3_ is generally more tolerated for efficacy compared with other substituents. Compound **25b,** endowed with this substituent, possessed higher selectivity for the CQR strain over the CQS strain, implying a higher resistance index. Furthermore, preliminary evaluation of the compounds for their potential binding affinity to hematin, which is a common target for antimalarial drugs, suggests a mechanistic modality independent of the hemozoin inhibition pathway.

Encouraged by the promising biological performance of the 2-phenylbenzimidazole Ir(III)Cp* complexes (**25**) against *P. falciparum* strains, a follow-up study entailed conjugation of a 4-aminoquinoline motif to either 2-phenylbenzimidazole or 2-pyridylbenzimidazole, and subsequent complexation, to yield neutral and cationic IrCp* and RhCp* complexes (Figure 6) [32]. The *C^N*-coordinated neutral complexes (**26a**–**c**) were more potent than their *N^N*-complexed cationic counterparts (**27a**–**c**) against both the K1 and NF54 strains of *P. falciparum*. Interestingly, the 2-phenylaminoquinoline-benzimidazole hybrid complexes (**26**) showed an improvement in activity compared with their corresponding 2-phenylbenzimidazole congeners (**25**) against the CQR K1 strain. Moreover, only the Me- and CF_3_-functionalized cationic derivatives (**27b** and **27c**) were superior to their corresponding non-quinolyl containing counterparts (**25c** and **25b**) against the CQS NF54 strain [31,32]. Furthermore, the new quinolinyl complexes demonstrated a significant binding affinity for synthetic hemozoin, β-hematin, exceeding that of the positive control drug, CQ. These findings indicate that the introduction of the pharmacophoric quinoline moiety is vital for antiplasmodial activity, as attested to by the results of the biological assays and the remarkable hemozoin binding activity. Once again, the examination of the substituent effects on activity revealed that CF_3_ enhances antiplasmodial efficacy. Finally, the neutral *C^N*-cyclometallated complexes (**26**), which displayed the most promising activity toward the *Plasmodium* strains, revealed increased selectivity toward parasitic cells over the CHO mammalian cells, with selectivity indices of >50 observed for complexes **26a** and **26b**.

## 3. Ruthenium Complexes

**Figure 7:**
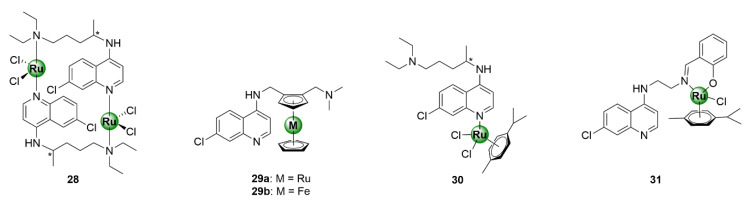
Ruthenium complexes based on the chloroquine scaffold [38,39,40,41,42].

Ruthenium is one of the most versatile metals within the PGM block, with a myriad of applications that span many industries, ranging from catalysis, solar energy technologies, cell-labelling utilities, and various therapeutic applications [33,34,35,36,37]. The success of ruthenium in catalysis is exemplified by the epochal discovery of the Nobel Prize-winning Grubbs catalyst for olefin metathesis [33]. In the context of biological applications, ruthenium complexes predominantly consist of η^6^-arene organometallic fragments and have been exhaustively explored in the field of oncology, as anticancer agents, with diverse modes of action. Evidence of the antimalarial activity of ruthenium-based compounds goes as far back as 1996 and has been demonstrated by several research groups, upon complexation of ruthenium to the chloroquine scaffold, which generated antiplasmodial agents with activity superior to chloroquine, against both CQR and CQS *P. falciparum* strains (Figure 7, **28**–**31**) [38,39,40,41,42]. Most notable is the ruthenium analogue, **29a**, of the ferrocenyl antimalarial clinical candidate ferroquine (**29b**). The incorporation of a bis(η^5^-cyclopentadienyl)ruthenium motif (ruthenocene), in place of ferrocene, has proven to be invaluable in unveiling the mode of action of ferroquine, in erythrocytes, owing to the enhanced amenability of ruthenium for cell-labelling compared with iron [43].

### 3.1. Tamoxifen Derivatives

**Figure 8:**
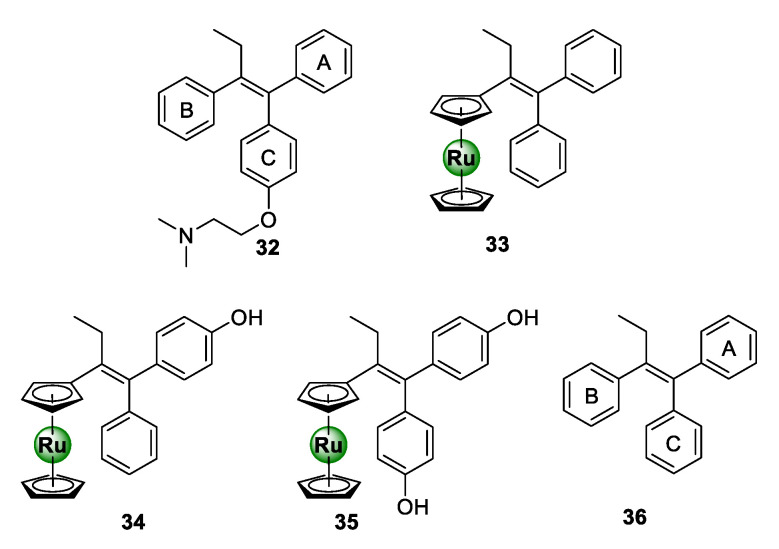
Chemical structures of tamoxifen (**32**), a tamoxifen-like compound (**36**), and its ruthenocenyl derivatives (**33**–**35**) [44].

Ru-arene complexes of bioactive motifs, such as thiosemicarbazones (TSCs), tetraoxanes, and salicylaldimines, have been shown to possess antiplasmodial activity over the years. Krettli and colleagues coordinated a ruthenocenyl entity into the scaffold of a tamoxifen-like compound (**36**), which displays structural similarities to the anticancer drug, tamoxifen (**32**) [44]. By replacing ring B with a ruthenocene entity, novel ruthenocenyl derivatives (**33**–**35**) were synthesized, which displayed antiplasmodial activity against the CQR W2 strain of *P. falciparum*, with IC_50_ values between 5.9 and 16.5 μM (Figure 8) [44]. Most notably, the ruthenocenyl derivatives were superior in activity to the parental organic ligand (**36**), which were shown to be inactive against the tested strain. This further supports the potential of metal incorporation to not only improve the biological activity of bioactive organic scaffolds, but also confer new biological activities.

### 3.2. Quinoline-Salicylaldiminato/Imidazole Ligands

**Figure 9:**
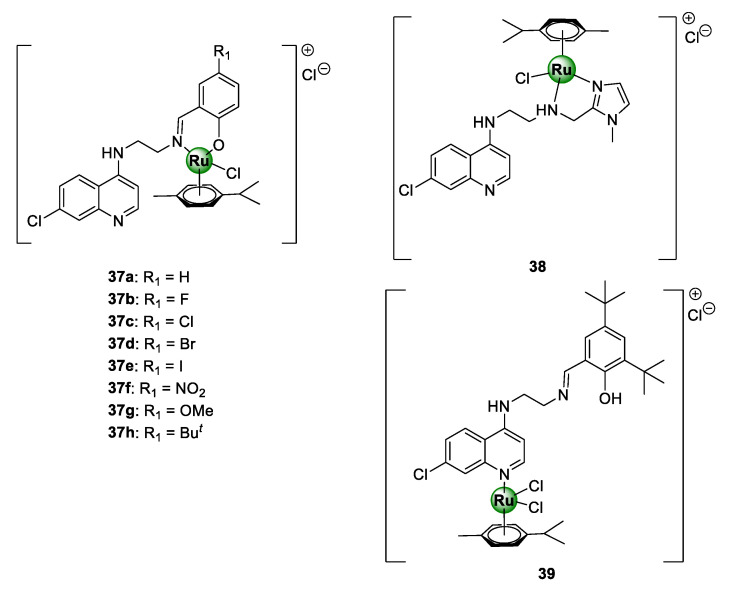
Quinoline-based Ru(II)(*p*-cymene) complexes, containing salicylaldimine (**37**) and imidazolemethylamine (**38** and **39**) derivatives [45].

Ekengard et al. reported on a series of quinoline-based ruthenium complexes, endowed with *N^O*-salicylaldimine (**37a**–**f**) and *N^N*-imidazolemethylamine (**38**) moieties [45]. These complexes were shown to inhibit the growth of CQS NF54 and D10 as well as CQR Dd2 strains of *P. falciparum*, with no indication of cross-resistance (Figure 9) [45]. SAR studies of the salicylaldimine complexes (**37a**–**f**) revealed a trend between the substituents and the antiplasmodial activity. This study suggested that an increase in the electron-withdrawing nature of the substituent, i.e., F > Cl > Br > I and OMe > H > NO_2_ > Bu*^t^*, resulted in an increase in the potency of the resultant complex. Furthermore, the replacement of salicylaldimine with a 2-imidazolemethylamine ligand (**38**) led to a drastic decrease in activity, by 8- to 10-fold. Intriguingly, the activity against both strains was significantly augmented upon complexation of the Ru(II)(*p*-cymene)Cl_2_ motif to the quinoline nitrogen atom (**39**).

### 3.3. Thiosemicarbazone and Organosilane Derivatives

**Figure 10:**
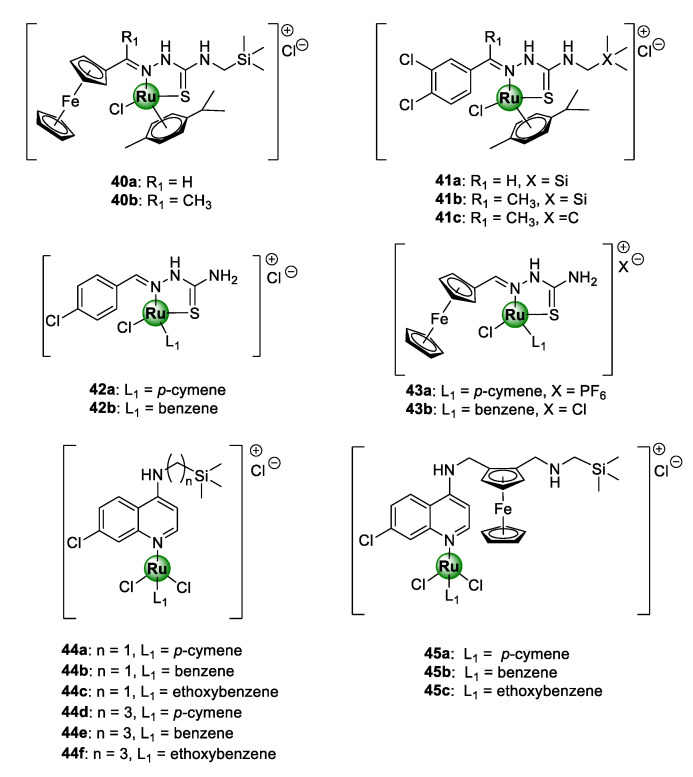
Chemical structures of Ru(II)-based thiosemicarbazone (TSC) (**40**–**43**) and aminoquinoline (**44**–**45**) complexes, possessing inhibitory activity against NF54 and Dd2 *P. falciparum* strain [46,47,48,49].

Heteronuclear Ru(II)(*p*-cymene) complexes, containing ferrocenyl (**40a**,**b**) and 3,4-dichlorophenyl (**41a**–**c**) bioisosteric units, based on an *N*-terminated organosilane TSC backbone, have also been reported (Figure 10) [46]. The appeal of ruthenium-based TSC complexes (**42**–**43**), as promising antiplasmodial agents, has been previously established by Adams et al. against CQS NF54 and CQR Dd2 strains of *P. falciparum* [47]. Within this study, the chloro-aryl thiosemicarbazones (**42a**,**b**) emerged as the most potent in the series [47]. In other studies, an organosilane moiety was incorporated into the lateral alkyl side chain of chloroquine- and ferroquine-derived ruthenium complexes, leading to the attainment of highly potent antimalarial agents (**44**–**45**), active in the low nanomolar range, targeting the malarial hemozoin pathway [48,49]. Organosilane moieties are famous for their exceptional lipophilic properties and have been incorporated into privileged drug scaffolds, to augment their biological activities and impart new therapeutic benefits [50,51]. This has been successfully applied in drug discovery to increase the cell permeability of drug molecules and, consequently, their tissue penetrating ability and thus bioavailability [52,53]. It should also be noted that all the silicon-containing aminoquinoline complexes (**44**) displayed lower resistance indices than CQ, with complexes **44d** and **44e** showing activity greater than CQ against the Dd2 strain of the parasite *P. falciparum* [48]. Furthermore, all of the ferroquine-derived counterparts (**45a**–**c**) were shown to be approximately 2.5-fold more potent than CQ against the Dd2 strain, and up to 8.5-fold more potent than ferroquine against the NF54 strain [49]. These observations were an unequivocal indication that the presence of the terminally appended organosilane motif had a significant impact on the antiplasmodial activity.

Premised on the promising antimalarial activity of Ru-TSC complexes and the beneficial organosilane motif, Smith and colleagues rationally combined TSC and organosilane moieties to produce *N^S*-chelated silyl TSC Ru(II) complexes **40**–**41** [46]. Despite the complexes showing high selectivity for the malaria parasites over the mammalian CHO cells, no significant improvement in activity was noticed upon complexation of the corresponding ferrocenyl and phenyl TSC ligands, as the resulting Ru(II) complexes exhibited lower, albeit comparable, toxicity to the ligands. An exception to this was the non-silyl 3,4-dichlorophenyl representative complex **41c**, which was 68 times more active than its respective ligand against the CQS NF54 strain. On a positive note, these new silyl complexes were significantly more active than their related non-silyl analogues (**42**–**43**) that had been reported earlier [47]. Again, this was further confirmation of the pharmacological importance of the organosilane moiety appended to the side chain of the TSC structural motif, as previously shown for chloroquine and ferroquine Ru(II) complexes [48,49]. Table 1 lists the IC_50_ values of complexes **40**–**45** against the CQS NF54 and CQR K1 strains of *P. falciparum*, along with the resistance and selectivity indices for selected compounds [46,47,48,49].

### 3.4. Quinoline-Trioxane Ligands

**Figure 11:**
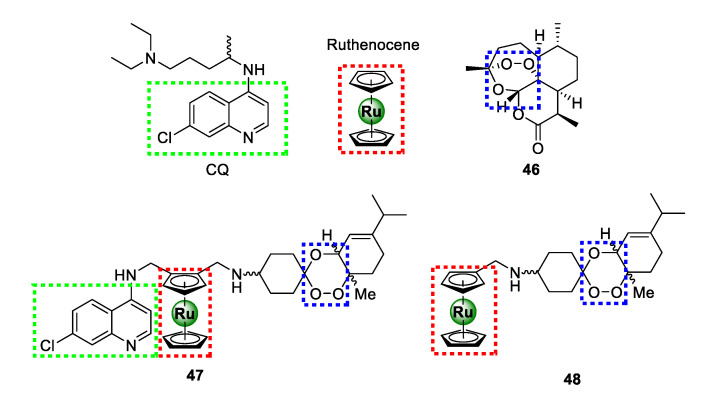
Chemical structures of highly potent antimalarial ruthenocene-quinoline-trioxane hybrids assembled from chemical motifs of chloroquine and artemisinin [54].

In 2016, Martinez and colleagues ingeniously amalgamated two antimalarial pharmacophoric entities, 4-aminoquinoline present in chloroquine (CQ) and 1,2,4-triaoxane of artemisinin (**46**), together with ruthenocene, culminating in a novel ruthenium-based antimalarial agent (**47**) endowed with three moieties that are vital for antimalarial activity (Figure 11) [54]. The resulting organo-ruthenium aminoquinoline-trioxane hybrid (**47**) was highly potent against CQR K1 (16.96 ± 2.93 nM) and Dd2 (51.16 ± 10.39 nM) strains, with >7-fold improvement in activity relative to CQ. Interestingly, this compound showed high selectivity for the parasitic strains over the healthy human MRC5 cells translating into marked selectivity indices of 92 and 30 for the K1 and Dd2 *P. falciparum* strains, respectively. The impressive efficacy of **47** was comparable to that of the other potent organometallic antimalarial compounds, such as ferroquine, ruthenoquine, and trioxaferroquine, poised to overcome clinical resistance by CQR *P. falciparum* strains. Preliminary SAR interrogation of the hybrid revealed that the 4-aminoquinoline nucleus is critical for activity as the non-quinoline ruthenocene-trioxane analogue **48** exhibited inferior activity to hybrid **47**. Moreover, the plain trioxane substrate (**46**) had even more diminished activity, thus underscoring the pharmacological importance of the ruthenocene unit.

### 3.5. Heteroaromatic Ligands

**Figure 12:**
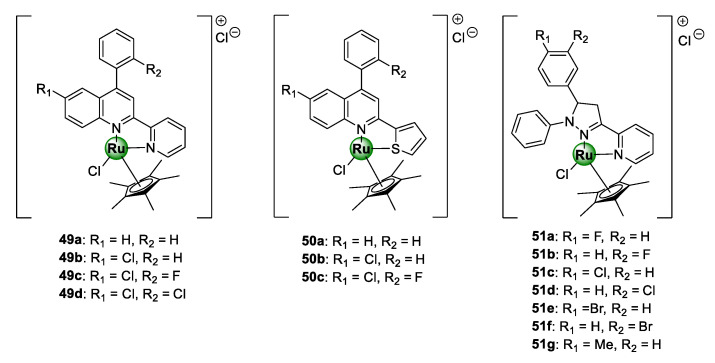
Antiplasmodial *N^N*- and *S^N*-chelated [Ru(III)Cl]Cp* complexes of 4-arylaminoquinoline scaffold *N^N*-coordinated pyrazoline congeners [55,56].

Patel et al. conjugated Ru(III)Cp* to a selection of planar polycyclic heteroaromatic ligands with structural conformations desirable for DNA binding, in order to target resistant clinical isolates of the *P. falciparum* parasite, as well as fungal (*S. bombe*) and bacterial pathogens (*S. Aureus*, *S. marcescens*, *B. subtilis*, *P. aeruginosa,* and *E. coli*) [55,56]. The first cohort of complexes (Figure 12) was assembled from a modified 4-arylquinoline moiety grafted with heteroaryl ring units, i.e., 2-pyridine and 2-thiophene, at C2 to produce *N^N*- and *N^S*-coordinated Ru(III)Cp* complexes (**49a**–**d** and **50a**–**c**), respectively [55]. All the complexes showed activities below 1.0 mg/mL with higher potency being observed for the *N^N*-chelated pyridyl congeners (**49a**–**d**) over the thiophenyl counterparts (**50a**–**c**). On the other hand, the antiplasmodial effects of the corresponding uncomplexed ligands were inferior (around 1.6 mg/mL), once again stressing the medicinal benefits of incorporating metallic complexes into bioactive scaffolds as a viable avenue to improve their antimalarial activity. Preliminary toxicity assessments of the compounds in brine shrimp indicated strong cytotoxicity of complexes, which could be interpreted as a promising indication of their antiproliferative potential as anticancer agents. 

Like cisplatin, ruthenium complexes possess a significant affinity for binding to nucleic acid (DNA or RNA) through a different mechanism, leading to the disruption of several essential biological processes vital for the survival of the targeted pathogen, which subsequently induces cell death [57,58]. Consequently, elucidation of a probable mechanism of action of the complexes (**49**–**50**) was carried out on herring sperm DNA (HS-DNA) using various biochemical protocols: UV/vis titration, viscosity (or hydrodynamic volume) measurements, agarose gel electrophoresis (AGE), and in silico docking simulations [55]. The authors demonstrated that the complexes exhibit strong DNA-binding affinity via intercalation with binding constants in the range 0.30 × 10^5^–6.25 × 10^5^ L/mol. Intriguingly, compound **49c**, which emerged as the most toxic candidate against *P. falciparum* parasitaemia in the study, displayed the highest affinity for HS-DNA, thus suggesting a correlation between activity and DNA interaction. The second cohort of polycyclic [Ru(III)ClCp*] complexes, featuring tri-arylated pyrazoline ligands (**51a**–**g**), was also active against the clinical isolates in the IC_50_ range of 0.54–2.15 mg/mL, with compound **51a** exhibiting the highest activity [56]. The complexes interacted with DNA in a similar manner to the quinolinyl complexes **50**–**51** and the binding constants were in the same order of magnitude.

### 3.6. Quinoline/Benzimidazole Ligands

**Figure 13:**
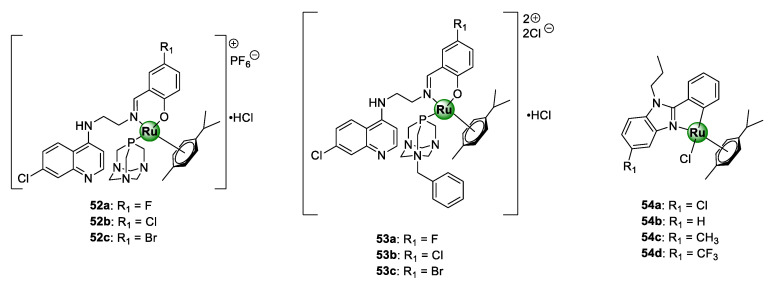
Quinolinyl ruthenium(II)-arene PTAs (RAPTAs) **52**–**53** and 2-phenylbenzimidazole Ru(II)(*p*-cymene) complexes **54a**–**d** with antimalarial activity [26,31].

A study by Stringer et al. included quinolinyl RAPTAs **52**–**53** showing inhibitory activity on the growth of CQS NF54 and CQR K1 *P. falciparum* strains at half-maximal concentrations in the ranges of 0.10–0.40 and 1.6–4.5 μM, respectively (Figure 13) [26]. Generally, incorporation of the PTA moiety by coordination to the metallic centre of the Ru(II)(*p*-cymene) unit augmented the activity of the complexes against the sensitive strain for the same reasons discussed earlier for the Ir(III)Cp* congeners. High selectivity indices of >10 were observed for the malaria parasites over the mammalian cells for selected promising complexes tested on CHO cells. Antiplasmodial activity of neutral Ru(II)(*p*-cymene) derivatives **54a**–**d** (Figure 13), of the 2-phenylbenzimidazole PGM complexes, was also reported in the same range as their Ir(III)Cp* congeners **25a**–**d** (Figure 6) discussed earlier [31]. Likewise, the activity of these complexes was superior to the uncomplexed ligands **24a**–**d** (Figure 6), emphasizing the significance of the presence of the organometallic Ru(II)(*p*-cymene) unit.

## 4. Rhodium Complexes

The application of rhodium complexes, especially the forms with the 3+ oxidation state, i.e., Rh(III), for biological evaluation has often been overlooked because of the perception that they may show limited biological activity owing to their inert chemical nature [59]. However, recently, rhodium complexes have been increasingly gaining favour among medicinal organometallic chemists to expand the antimalarial drug arsenal and potentially diversify the mechanisms of action. An early example of an antimalarial rhodium complex is the Rh(I)-CQ conjugate of **2** (Figure 1) formed by coordination of a RhCl(COD) unit (COD = 1,5-cyclooctodiene) via the N1 atom of the quinoline nucleus, which demonstrated enhanced activity compared with the control drug in vivo, in mice infected with *P. berghei* parasitemia [38].

### 4.1. Salicylaldiminato Ligands

**Figure 14:**
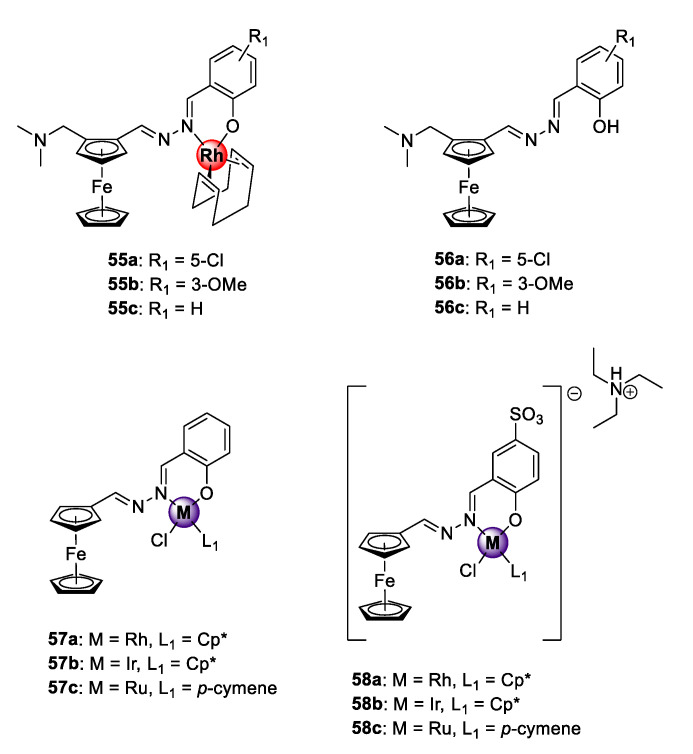
Bimetallic organometallic azines with antiplasmodial activity [23,60,61].

A series of heteronuclear ferrocenyl azine complexes **55a**–**c** was endowed with the organometallic Rh(I)COD motif via bidentate *N^O*-coordination, which was active against CQS NF54 and CQR K1 *P. falciparum* strains (Figure 14) [60]. The complexes showed antiplasmodial activities in the low micromolar range against both strains and low resistance indices for **55b**–**c** (RI = 0.22–0.85). The uncomplexed ferrocenyl azine ligands **56a**–**c** were less potent, signifying the favourable pharmacological effects of introducing the organometallic Rh(I)COD unit. Mechanistic examination using the NP40-mediated β-hematin assay suggests disruption of the heme detoxification pathway as a possible mode of action. Before these studies, Rh(I)ClCp* congeners **57a** and **58a** exhibiting reversible redox character were investigated for their antiplasmodial effects on the CQS NF54 parasites along with their Ir(III)ClCp* (**57b** and **58b**) and Ru(II)Cl(*p*-cymene) (**57c** and **58c**) derivatives [23,61]. In both studies, the Rh(I)ClCp* complexes displayed comparable activities to the Ir(III)ClCp* and Ru(II)(*p*-cymene) derivatives, placing the rhodium metal as an attractive alternative to the extensively studied iridium and ruthenium metals for the generation of bioactive organometallic compounds. Again, the bimetallic complexes were significantly superior in activity to their respective ferrocenyl ligands. Introducing aqueous solubility to complexes **58a**–**c,** by incorporation of a water-soluble sulfonate group at position C5 of the benzene ring, led to a decrease in the activity of the complexes, and no discernible β-hematin binding affinity was observed at the maximum tested concentration (IC_50_ > 100 μM). The latter observation would seem to suggest that the observed inhibitory action of the water-soluble sulfonate compounds on the screened strain proceeds via alternative modes of action such as the generation of reactive-oxygen species (ROS) in which the ferrocene unit plays a pivotal role by undergoing reversible Fenton-type redox processes inside the DV of the malaria parasite [62,63].

### 4.2. Quinoline-Salicylaldiminato and Sulfadoxine Ligands

**Figure 15:**
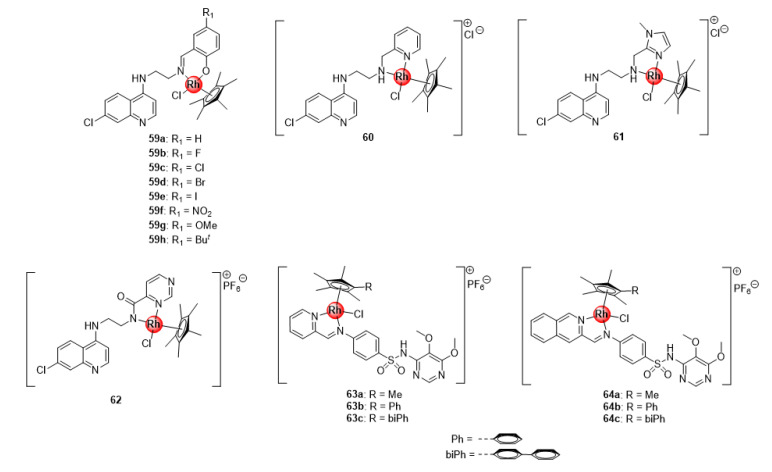
Antiplasmodial Rh(III) complexes based on chemical scaffolds of known antimalarial drugs, chloroquine (**59**–**62**) and sulfadoxine (**63**–**64**) [24,25,30].

In addition to the bidentate chloroquine-derived *N^N*- and *N^O*-chelated Ir(III)ClCp* complexes **7**–**10** (Figure 2), Nordlander and colleagues reported the antiplasmodial activity of Rh(III)ClCp* congeners **59**–**62** exhibiting activity against CQS NF54 and CQR Dd2 *P. falciparum* strain (Figure 15) [24,25]. These rhodium complexes were more active than their Ir(III)ClCp* variants **7**–**10** in the sub-micromolar range (IC_50_: 0.16–0.209 μM) against the sensitive strain, although less active against the resistant strain (0.20–0.40 μM). Interrogation of complexation effects and influence of substitution effects on the benzene ring revealed similar SAR trends as the iridium derivatives (**7**–**10**) previously discussed. Likewise, Chellan et al. also studied the antiplasmodial potency of the rhodium(III) counterparts (**63**–**64**) of the organometallic iridium(III) pyridyl- and isoquinolyl-sulfadoxine conjugates (**22**–**23**, Figure 5) against CQS 3D7 and NF54 strains, as well as resistant Dd2 parasites, which generally displayed a significant improvement in activity relative to the Ir(III) complexes across all strains (Figure 15) [30]. Again, the generated SAR trends were similar for both types of complexes, as discussed earlier. The results from the studies by these research groups seem to suggest that the oxidation state of the metallic centre is critical in regulating the activity of the rhodium complexes; particularly, the 3+ oxidation state of the rhodium metal confers overall favourable antimalarial activity to the resultant organometallic agents, while the 1+ state is less favourable.

### 4.3. Thiosemicarbazone and Benzimidazole (Hybrid) Ligands

**Figure 16:**
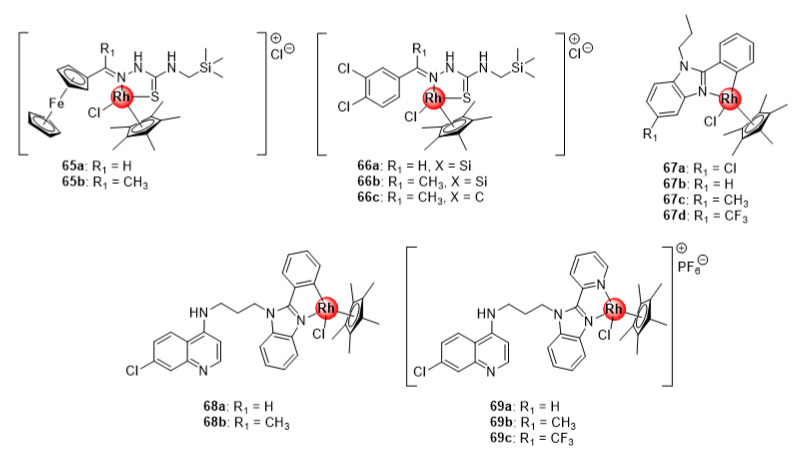
Rhodium(II) complexes of silyl ferrocenyl and 3,4-dichlorophenyl TSC, benzimidazole, and chloroquine-benzimidazole hybrid scaffold [31,32,46].

Rh(III)ClCp* silyl congeners (**65**–**66**) of Ru(II)(*p*-cymene) ferrocenyl and 3,4-dichlorophenyl TSC complexes (**40**–**41**, Figure 10) were also screened for their antimalarial potential against CQS NF45 and CQR Dd2 parasitemia in vitro, with the majority exhibiting better activity compared with the ruthenium(II) analogues (Figure 16) [46]. The activity of the Rh(III) compounds was correlated to their β-hematin inhibition activity. Intriguingly, the carbosilane complex **66b** was the most active in the series and exhibited better β-hematin activity than the control drug, chloroquine. This could be explained by the high lipophilic character of the silyl motif, which might enhance the binding interactions of **66b** to the growing hemozoin polymer chain. Also noteworthy were the minimal instances of cross resistance and general toxicity observed for these complexes, as indicated by their low resistance indices when comparing activities on both strains and high selectivity indices on the mammalian CHO cell line. Similarly, Rh(III)ClCp* benzimidazole complexes (**67**) inhibited the growth of sensitive strains of *P. falciparum*, albeit less effective than the Ru(II)Cl(*p*-cymene) (**54**, Figure 13) and Ir(III)ClCp* (**25**, Figure 6) derivatives [31]. Conjugation of these complexes to the 4-aminoquinoline nucleus significantly enhanced the activity of the compounds (**68**–**69**), making them more potent than the Ir(III) congeners with higher selectivity towards the malaria parasites (NF54 and K1) over the mammalian CHO cells (SI > 20) [32]. Collectively, data from these studies seem to reinforce the previous observation regarding the beneficial pharmacological effects of the +3 oxidation state on the antiplasmodial activity of Rh(III) complexes.

### 4.4. Quinoline-Polyamine Scaffolds

**Figure 17:**
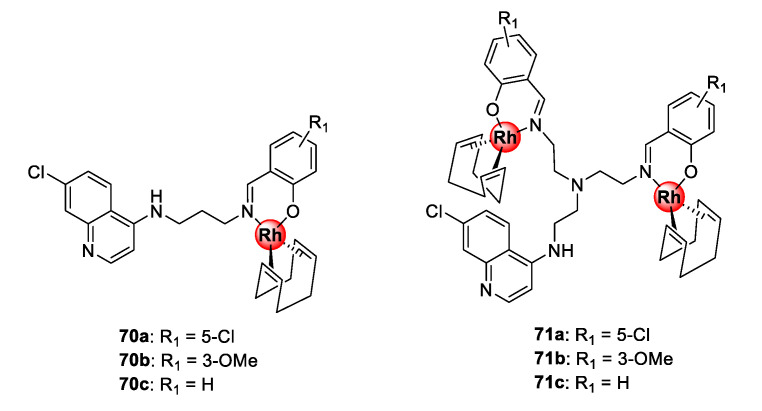
Polyamine quinolinyl Rh(I)COD complexes showing inhibitory activity towards sensitive and resistant *P. falciparum* parasites [64].

Bimetallic polyamine quinolinyl Rh(I)COD complexes (**70**–**71**) assembled by *N^O*-coordination via the terminal salicylaldimine motif were demonstrated to possess inhibitory effects towards the growth of sensitive and resistant *P. falciparum* strains (Figure 17) [64]. Compounds **71a**–**c**, with shorter ethyl linkers, displayed higher selectivity for the resistant parasite strain over the sensitive strain, while the mononuclear complexes, **70a**–**c**, endowed with a propylamine spacer, had lower resistance indices. Interestingly, compounds **71a**–**c** demonstrated a higher binding affinity for synthetic hemozoin than chloroquine by almost two-fold and, in all cases, the complexes were more effective than their respective metal-free ligands. This further validates the strategy of incorporating organometallic complexes into known drug scaffolds as a viable approach to modulate their activity.

## 5. Osmium Complexes

**Figure 18:**
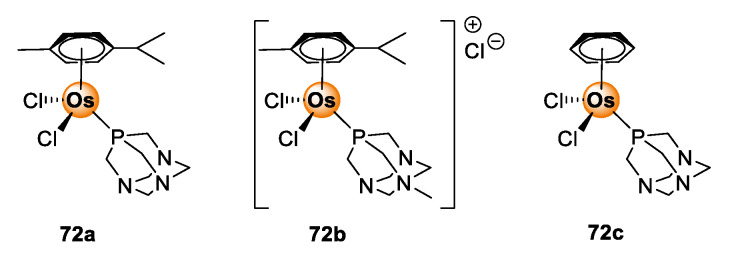
Chemical structures of Os(II/III) RAPTA analogues with anticancer activity [72].

Osmium poses as another interesting candidate from the PGM block for biological application. Osmium complexes with oxidation states 2+, 3+, and 4+ or higher are well-documented in the literature [65]. The occurrence of various oxidation states of osmium enables the fine-tuning of redox properties of the complexes for the regulation of redox-dependent biological processes, such as the generation of ROS, within the pathogenic cells of the targeted disease [66]. Furthermore, the choice of coordinating and ancillary ligands allows further tuning of photophysical properties and confers three-dimensional spatial configuration to the complex, which offers better recognition and guest–host interaction with biological receptors [67]. As a result, osmium complexes have become versatile with great promise for utility in cancer treatment, including light-activated cancer therapy and photodynamic therapy [68,69]. The anticancer effects of osmium complexes are succinctly discussed in the reviews by Hanif et al. [70], Meier-Menches et al. [71], and Nabiyeva et al. [72]. Examples of osmium complexes that demonstrate antiproliferative effects include the Os(II/III) analogues (**72**) of the well-studied Ru(II/III) RAPTA complexes shown in Figure 18 [72].

### Quinoline-Salicylaldiminato/Imidazole Ligands

**Figure 19:**
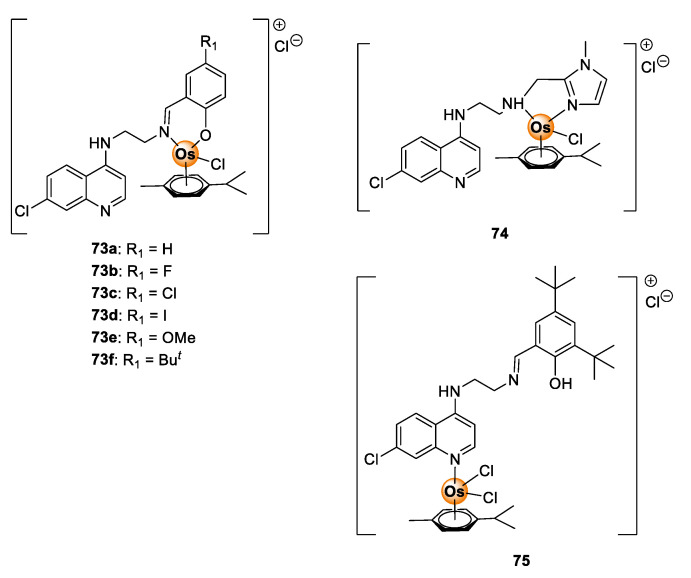
Quinolinyl Os(II) complexes possessing antiplasmodial activity against sensitive and resistant *P. falciparum* strains [45].

Despite the demonstrable biological potential of osmium complexes, antimalarial investigation of osmium complexes is limited in the literature. To date, only one study investigating the antimalarial activity of osmium complexes is known. In this report, Nordlander and colleagues evaluated Os(II) analogues (**73**–**75**) of Ru(II)Cl(*p*-cymene) complexes **37**–**39** (Figure 9) in vitro on sensitive (NF54 and D10) and resistant (Dd2) *P. falciparum* strains for antiplasmodial activity (Figure 19) [45]. The activity of the complexes **73**–**75** was comparable to the Ru(II) analogues **37**–**39** on all tested strains. In a few cases, the Os(II) complexes were more potent than their Ru(II) counterparts, signifying the attractive antimalarial potential of osmium-based compounds. Given these observations, it is apparent that osmium complexes are worthy of further consideration in the context of their antimalarial activity.

## 6. Platinum and Palladium Complexes

**Figure 20:**
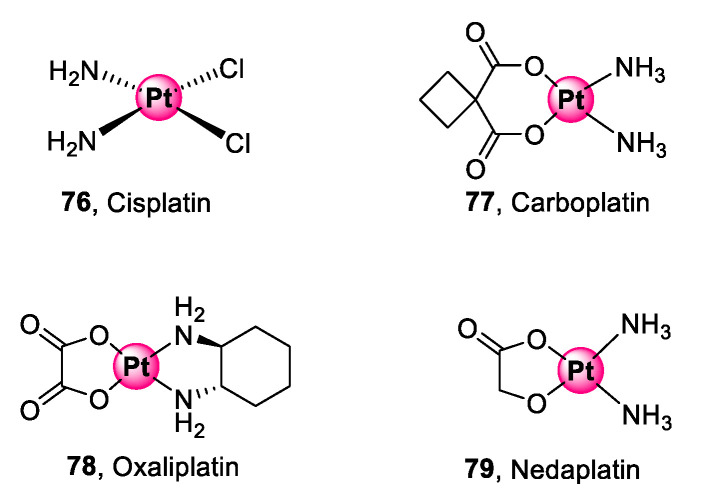
Next generation platinum(II) complexes approved for cancer treatment [73,74,75].

Platinum is the pioneering metal in the interest of PGM complexes as biological agents for targeting diseases. The activity of platinum complexes goes as far back as the 1960s with the epochal discovery of the anticancer activity of *cis*-diamminedichloridoplatinum(II), commonly known as cisplatin, by Rosenberg and co-workers [73]. Next generation platinum(II) derivatives (**77**–**79**), shown in Figure 20, have since been developed and approved to address the challenges faced by cisplatin (**76**), namely, limited selectivity, development of clinical resistance, and acute toxicity [74,75].

**Figure 21:**
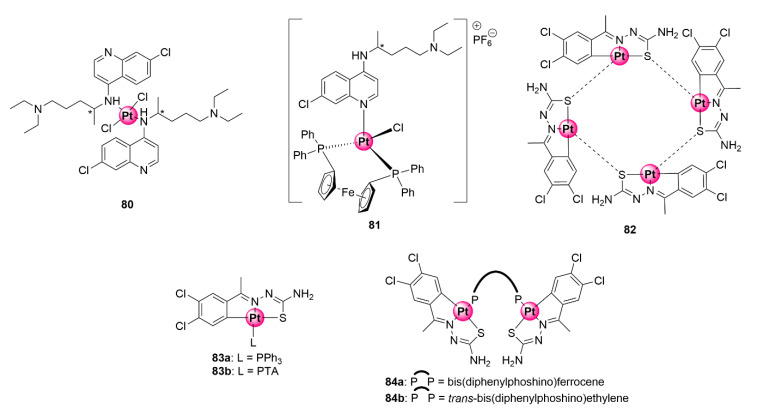
Inorganic (**80**–**81**) and organometallic (**83**–**84**) platinum complexes with antimalarial activity [20,79,80].

Numerous examples of inorganic platinum complexes possessing antiplasmodial activity have been reported in the literature. Several reviews [19,76,77] and a published book chapter [78] give detailed accounts of antiplasmodial platinum-based complexes. Representative examples of inorganic platinum complexes **80**–**81** with antimalarial activity are also illustrated in Figure 21 [20,79]. In many cases, coordination of ligands, particularly known organic antimalarials, with the platinum metal leads to an improvement in antimalarial activity compared with the uncomplexed ligands. It is evident from these presented examples that platination may be an ingenious strategy to augment the efficacy of antimalarial agents, which is a crucial consideration in the fight against the plight of clinical resistance development. Notwithstanding, there is a limited account of cyclometallated platinum complexes, i.e., organometallic compounds with carbon to platinum (C-Pt) bonds, in the literature with antiplasmodial activity against *P. falciparum* strains.

### Thiosemicarbazone Ligands

Chellan et al. synthesized tridentate *C^N^S* cycloplatinated TSC complexes with antiplasmodial activity against CQS D10 and CQR Dd2 strains as well as antiproliferative and antitrichomonal effects on ovarian cell lines and *Trichomonas vaginalis* parasites (Figure 21) [80]. Cycloplatination was achieved via C-H activation of the TSC ligand to generate a tetrameric complex (**82**) with platinum-sulfur bridges. Ligation of this tetramer with mono- and bi-phoshino ligands (triphenylphosphine (PPh_3_), PTA, and bis(diphenylphosphino)ferrocene and *trans*-bis(diphenylphosphino)ethylene, respectively) led to the attainment of the corresponding mono- and binuclear cycloplatinated TSC products (**83a**,**b** and **84a**,**b**). Pharmacological screening of complexes for their antiplasmodial effects in vitro revealed that only the tetra- and mononuclear complexes (**82** and **83a**,**b**) were active against the plasmodial parasites, with IC_50_ activities between 19.93 and 32.92 μM, whereas the binuclear equivalents **84a,b** endowed with bis-phosphino ligands were impotent. Intriguingly, compound **83a** ligated with PPh_3_ showed no indication of cross-resistance, as attested by its higher selectivity towards the resistant Dd2 strain (14.47 ± 1.98 μM) over the chemosensitive D10 variant (19.93 ± 3.74 μM). The PTA analogue similarly showed minimal effects of cross-resistance because activities on both strains were almost equivalent (21.42 ± 1.22 μM vs. 24.90 ± 3.24 μM).

On the other hand, organometallic complexes of the related congener metal, palladium, are more prevalent in the literature. The coordination chemistry of palladium complexes closely resembles their platinum counterparts more than any other metals within the PGM block [81,82]. Moreover, the faster ligand-exchange and aquation rates (more than 10^5^ times) of palladium complexes lead to higher solubility relative to their platinum congeners, making them attractive for application in biological systems [83,84]. Hence, interest in their biological potential is only logical considering the success of the structurally similar platinum complexes in anticancer treatment. Consequently, there have been various studies on the biological activity of palladium complexes since Graham and Williams proposed the investigation of palladium complexes as anti-infective agents and promising anticancer alternatives to platinum in their pioneering studies [85]. Like in cancer, palladium complexes in malaria research have been explored in the literature with a fair representation of cyclopalladated organometallic variants [86,87,88,89,90,91].

**Figure 22:**
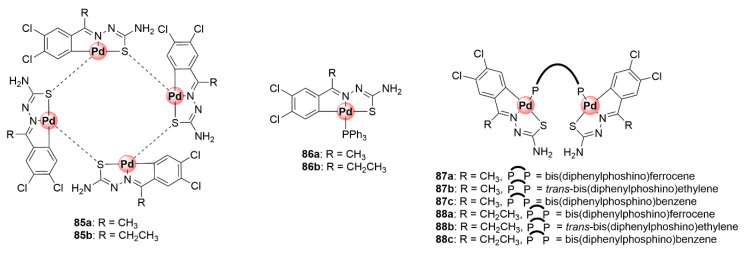
Chemical structures of antiplasmodial cyclopalladated TSC complexes [88].

Organometallic mono-, di-, and tetranuclear *C^N^S*-coordinated Pd(II) complexes based on the TSC scaffold were synthesized and studied for their antiplasmodial activity against CQS 3D7 and CQR K1 *P. falciparum* strains to ascertain the influence of coordination with palladium and the effects of having multiple metallic centres on the activity of the ligands [88]. Tetranuclear complexes **85a**,**b** were assembled by coordination of previously tested antiplasmodial TSC ligands with potassium tetrachloropalladate(II), forming a product with a Pd_4_S_4_ core due to the palladium-sulfur bridging bonds (Figure 22). Cleavage of the Pd_4_S_4_ bridges in these complexes with PPh_3_ and diphosphino ligands yielded mono- and binuclear cyclopalladated complexes (**86a**,**b** and **87**), respectively. The biological assessment of the obtained compounds revealed that incorporation of the Pd(II) centre increased the inhibitory activity of the complexes as the tetranuclear (**85a**,**b**) and mononuclear (**86a**,**b**) compounds were more potent than their respective TSC ligands against CQS 3D7 and CQR K1 strains, with IC_50_ values below 6 μM. The mononuclear complexes emerged as the most active in the study particularly against the sensitive strain, suggesting the presence of multiple Pd(II) centres had no marked effects on biological activity. Intriguingly, only the complexes containing the 1,1’-bis(diphenylphosphino)ferrocene linkage (**87a** and **88a**) were active, while the non-ferrocenyl ethylene and benzene counterparts were inactive within the binuclear series. This highlights the beneficial pharmacological effects of ferrocene [63].

**Figure 23:**
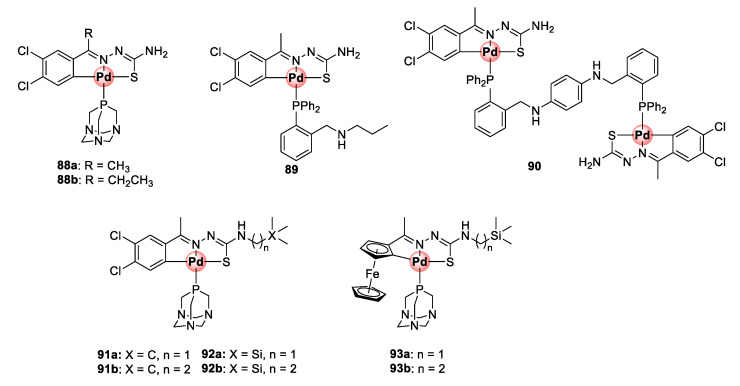
Antiplasmodial mono- and dinuclear cyclopalladated complexes and their organosilane derivatives [89,90].

A follow-up study incorporating PTA (**88a**,**b**) and 2-phosphinobenzylamine (**89**) coordinating ligands led to the identification of mononuclear cyclopalladated TSC complexes possessing improved antiplasmodial activities in the low micromolar range (1.59–2.69 μM) against CQS NF54 and CQR Dd2 strains, with low resistance indices often observed (Figure 23) [89]. Similar to the previous findings, the mononuclear complexes were superior in activity to their corresponding free TSC ligands, and the tetranuclear complexes (**85a**,**b**) were impotent on both strains. Expansion of the bisphosphino bridge with a bulky three-benzene linker for the binuclear complex **90** did not enhance the antiplasmodial effects of the resulting compound.

The next campaign sought to further augment the activity of mononuclear cyclopalladated TSCs by introducing a lipophilic organosilane motif into the backbone TSC structure of the complexes in an attempt to increase their chances of crossing cellular membranes and potentially retain them in the active site of the targeted malaria parasite (DV) (Figure 23) [90]. As previously discussed, the strategy of incorporating organosilane motifs into drug molecules is touted for enhancing their lipophilicity and imparting beneficial therapeutic properties such as enhanced tissue penetration and cell permeability. The dichlorobenzene motif and coordinating PTA moieties were retained (**91a**,**b**), and one- and three-carbon silyl linkers were appended to the terminal NH_2_ group of the TSC backbone (**92a**,**b**). Two ferrocene-cyclopalladated representatives (**93a**,**b**) were included in the study by replacing the dichlorobenzene unit. Like in the previous studies, cyclopalladation greatly enhanced the inhibitory activity of the complexes by an average of ~31-fold relative to the uncoordinated ligands on the NF54 and Dd2 strains. Ferrocenyl complexes **93a**,**b** active between 1.07 and 1.52 μM were less potent than the dichlorobenzene derivatives, which exhibited activities mainly in the sub-micromolar range (0.29–0.88 μM) against both strains. Most noteworthy, these complexes showed a great improvement in activity compared with the variants devoid of the one- and two-carbon linkers on the terminal carbon from the previous study (**88a**,**b**), which were only active at 1.73–2.69 μM on the same parasites. To further illustrate the pharmacological significance of the organosilane moiety, when the authors compared the effectiveness of the silyl complexes (**92a**,**b**) to their carbon analogues (**91a**,**b**), the activity was found to be twice as good with preferential activity towards the resistant strain (Dd2). It is clear from these observations that the incorporation of the organosilane moiety is a practical approach to enhance the therapeutic efficacy of antiplasmodial agents, as also observed elsewhere in other studies.

## 7. Insights into Antiplasmodial Mechanisms of Action of Organometallic PGM Complexes

Extensive studies have been directed towards evaluating the inhibitory activity of PGM-derived organometallic complexes as potential antimalarial agents against a myriad of *P. falciparum* strains. The same could be said for anticancer research, which has by far received much greater attention than antimalarial research. To this end, there has been a hive of activity among bioorganometallic chemists not only to demonstrate the biological activity of such complexes with cell-based assays, but also in an attempt to elucidate their possible modes of action by which they may exhibit activity. This is a crucial step in the early stages of the drug development pipeline and raises the chances of a drug candidate making it through the clinical assessment phases. For instance, the mode of action of anticancer complexes such as cisplatin and related analogues has been established through thorough mechanistic investigations [92,93]. However, the same cannot be said for a majority of antimalarial organometallic complexes. In this section, we draw attention to the literature reports of this sparsely-researched field and the available data on the investigation of possible mechanistic modalities of organometallic PGM complexes and potential opportunities for further exploration of such targets. Mechanistic insights into the antimalarial organometallic complexes have primarily focused on three targets, namely, hemozoin inhibition (a mainstay for quinoline antimalarials); interaction with nucleic acids, particularly DNA; and intracellular catalysis. These are discussed below.

### 7.1. Blocking the Plasmodial Heme Biocrystallisation Pathway

During the blood stages of the malaria parasite’s lifecycle, the *Plasmodium* species digests haemoglobin from the human host to support its metabolic requirements and for sustenance [94]. The process leads to the concomitant formation of free haem molecules that are toxic to the parasite through the production of reactive oxygen species, leading to its death. To counteract this effect, the malaria parasite has developed a pathway that biocrystallizes free heme molecules into insoluble, non-toxic crystalline hemozoin, also known as the malaria pigment. This takes place inside the parasite’s acidic DV (pH 5.2–5.6). Traditional antimalarial drugs, most notably quinolines, have been proposed to induce antimalarial activity by blocking this pathway via binding interaction with heme [95,96]. For instance, chloroquine accumulates in the plasmodial DV and binds to polymerizing heme complexes to halt the biocrystallization process, thereby liberating more heme molecules to kill the parasite [96]. It is on this premise that this pathway has been a subject of widespread research interest to devise novel bioactive compounds for antimalarial activity through its blockade. Organometallic complexes of transition metals, such as ferroquine, have been successfully applied to target heme detoxification [97,98]. Apart from this, several organometallic complexes show reversible redox character capable of generating ROS due to the presence of the metallic centre and can also introduce new therapeutic benefits, including high lipophilicity and improved cell membrane permeability [99].

Several antiplasmodial PGM organometallic complexes have been explored for the inhibition of the hemozoin pathway with selected iridium [26,29,32], ruthenium [46,48,49], and rhodium [32,46,60] complexes already discussed in this review showing positive inhibitory activity. These were investigated employing the detergent-mediated β-hematin bioassay developed by Ncokazi and Egan, an invaluable tool in detecting and measuring inhibition of the plasmodial heme biocrystallization process by antimalarial agents [100]. Although many complexes evaluated for the blockade of this pathway were less potent than the clinical antimalarial drug, chloroquine, their β-hematin inhibition activity was in agreement with their antiplasmodial effects against the tested *P. falciparum* strains in most cases. Interestingly, compounds containing the organosilane moiety in their scaffolds such as the Rh(III)Cp* TSC, **66b**, as well as ferroquine- and chloroquine-derived Ru(II), Rh(II), and Rh(I) complexes, were superior hemozoin inhibitors to the control drug (CQ) [46,48,49]. However, a direct correlation between antiplasmodial activity and hemozoin inhibition could be discerned for the quinolinyl complexes [48,49].

### 7.2. Targeting DNA Interaction for Antiplasmodial Activity

DNA is integral in an array of biochemical processes, from the coding of genes necessary for regulation of normal cellular functions to the expression of proteins that are pivotal in the pathology of diseases. Considering the substantial variations between human and plasmodial genomes, particularly nucleotide base composition, it is possible and sensible to devise chemical agents that target the malarial parasites over normal human cells by exploiting these differences. Most noteworthy in these variations is the abnormal adenosine- and thymine-rich content in the DNA of *Plasmodium* parasites [101,102]. This alteration affects the structure of plasmodial DNA and potential binding sites for external compounds, offering medicinal chemists an opportunity to formulate chemotypes that can selectively target these sites to inhibit the growth of *P. falciparum* parasites, as has been demonstrated in the literature [102,103,104]. Ruthenium organometallic complexes have been tested for DNA interaction as a possible mechanism of action for inducing antiparasitic effects.

**Figure 24:**
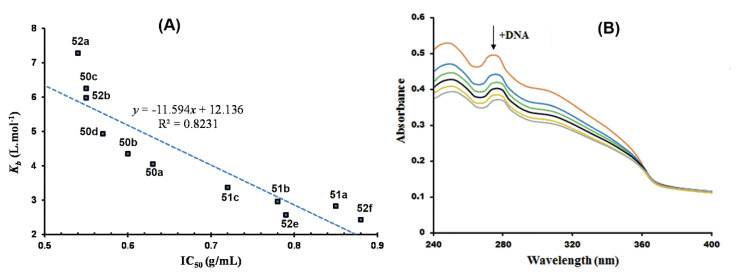
DNA binding affinity of Ru(III)Cp* complexes reported by Patel et al. [55,56]. (**A**) Scatter chart of DNA binding constants of the complexes plotted against their respective antiplasmodial activities. (**B**) UV/vis DNA titration curve of complex **49c**. Adapted from [55,56].

The Ru(III)Cp* complexes **49**–**51** (Figure 12) reported by Patel and associates were also investigated for DNA interaction using HS-DNA, as previously discussed [55,56]. Ruthenium(III) complexes are touted for their impressive DNA binding affinity as possible chemotherapeutic agents, and this has been extensively studied in cancer research of bioorganometallic complexes [105,106,107,108]. The binding affinity of the complexes, quantified in terms of binding constants, agreed with their observed in vitro antiplasmodial activity assessed in *P. falciparum* clinical isolates. This relationship is illustrated in Figure 24A, showing a plot of DNA binding constants as a function of the antiplasmodial activity. As can be seen in the figure, compounds’ low IC_50_ values translate to high DNA binding affinity in a nearly linear relationship. The bathochromic spectral shifts (Δλ = 2–4 nm) observed in the UV/vis DNA titration curves, shown in Figure 24B for complex **49c**, suggested stacking interactions of the complexes with DNA base pairs, i.e., DNA intercalation, which was confirmed by DNA viscosity measurements and in silico docking simulations.

Agarose gel electrophoresis (AGE) experiments suggested the complexes were also capable of inducing cleavage of supercoiled pUC19 plasmid DNA by hydrolytic or oxidative processes. Additionally, the computational docking simulation studies with selected candidates revealed that the complexes elicit preferential binding to AT-rich regions of the model DNA structure used as a receptor via van der Waals interactions. This finding demonstrates the practicality of targeting plasmodial DNA over the DNA of the human host for the generation of organometallic chemotypes with good selectivity towards the malaria parasites. To further illustrate this point, the authors tested the complexes for general toxicity effects in a brine shrimp model, an inexpensive first-line measure for screening the lethality of antiparasitic compounds, which could be attributed to the complexes having selective binding affinity for plasmodial DNA. The complexes exhibited higher inhibitory potency in the *P. falciparum* parasites vis-à-vis the brine shrimp, indicating high selectivity indices of the compounds for malaria.

### 7.3. Disruption of Plasmodial Biochemical Processes by Intracellular Catalysis

Transition metals are renowned for their catalytic activity in many chemical transformations. Similar chemical transformations and analogous processes effected by these metals such as transfer hydrogenation, oxidation, and reduction are known to also occur in biological systems where they are mediated by enzymes. It is no surprise that, in recent years, transition metals have begun to be explored for catalysis of chemical processes in biological systems [109,110,111]. This has inspired medicinal and bioinorganic chemists to devise innovative metal-based compounds capable of acting as intracellular catalysts in relevant biochemical processes that are pivotal in the normal functioning of pathogenic cells or organisms, hopefully leading to their disruption and eventual cell death to produce therapeutic effects [109,110,111]. Considering that many clinical drugs react in stoichiometric equivalents with their biomolecular targets, which may require high dosages to deliver desired therapeutic efficacy and potentially lead to severe side effects, the intracellular catalysis strategy offers many advantages because only small quantities of the catalytic metallodrug would be needed to attain the same therapeutic effect, thus translating to lower drug dosages and minimal side effects [111]. This approach is increasingly gaining attention in contemporary drug discovery and is showing encouraging results in cancer research [112,113,114,115]. In particular, examples of PGM complexes bearing ruthenium, osmium, and rhodium metallic centres have been demonstrated to catalyse transfer hydrogenation associated with their antiproliferative activity [112,113,114,115].

**Figure 25:**
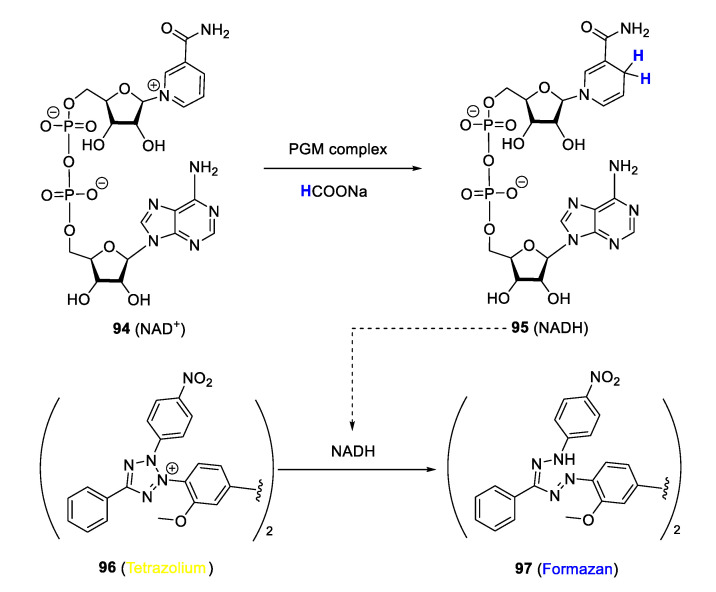
Monitoring of NAD^+^ conversion to NADH using a modified *Plasmodium* lactate dehydrogenase (pLDH) protocol. Adapted from [29,116].

In malaria research, targeting biochemical processes by intracellular catalysis is still nascent. However, there are representative PGM complexes in the literature, some of which are discussed in this review, reported to catalyse the transformation of biomolecules such as NAD^+^ and NADH, in an attempt to demonstrate their ability to perturb the associated biochemical process as a possible antiplasmodial mode of action. Studies by Melis et al. [29] and Stringer et al. [116] investigated the ability of quinolinyl Ir(III)Cp* and Rh(II)Cp* complexes to disrupt the transfer hydrogenation cycle of enzyme cofactors NAD^+^/NADH, a crucial biochemical process, via intracellular catalysis as a possible mechanistic modality to explain their antiplasmodial activity. The assay employed to realise this study is based on the principle of the *Plasmodium* lactate dehydrogenase (pLDH) method first reported by Makler and associates, which has become a mainstay for detecting the antiplasmodial activity of bioactive compounds in vitro [117]. In this protocol, NADH is utilised by pLDH to produce pyruvate from lactate while simultaneously reducing co-factor APAP^+^ into APAPH, which can convert a tetrazolium dye to blue formazan, thereby allowing monitoring of pLDH activity and growth of parasitaemia, as the two are closely linked. NADH can also reduce a tetrazolium dye to blue formazan in a similar manner to APAPH. In the modified procedure for detecting potential intracellular catalysis of the complexes, transformation of NADH to NAD^+^ in the presence of a catalytic PGM complex (and sodium formate as hydride source) was detected by following the absorbance of blue formazan formed from yellow tetrazolium dye in the reduction process (Figure 25). In all cases, the formation of blue formazan was detected, and the measured absorbance was proportionate to the concentration of complexes, suggesting positive catalysis of the NAD^+^/NADH transfer hydrogenation cycle by the complexes.

NMR experiments were conducted to corroborate the findings of the study using the pLDH model for NAD^+^/NADH monitoring. In this instance, NADH and a hydride source are incubated with catalytic amounts of the complex in a deuterated solvent and the disappearance or appearance of signals of NADH and NAD^+^, respectively, is monitored over time. The NMR spectra acquired after four hours of incubation showed new signals corresponding to NAD^+^ with no indication of any NADH signals present, thus confirming the successful conversion of NAD^+^ to NADH under the employed conditions. Furthermore, when parasites of the K1 resistant strain treated with complexes were co-incubated with increasing amounts of sodium formate, higher antiplasmodial activity was observed in a manner proportional to formate concentration. Collectively, these results provide clear evidence that the tested PGM complexes may indeed act as intracellular catalysts, capable of interfering with crucial biochemical processes, such as the NAD^+^/NADH transfer hydrogenation, in order to induce the antiparasitic effects as one of the modes of action, among others. The mechanism of catalysis is proposed to follow that explained in similar studies reported in the literature [118,119]. These findings show that the strategy of disrupting biochemical processes in malaria parasites via intracellular catalysis is a viable avenue for the search for innovative antimalarial drugs with novel modes of action. Notwithstanding, investigation of these complexes in intact plasmodial parasitic cells remains to be explored. More research is thus required to further elaborate the intracellular catalytic activity of these chemical entities and decisively link this property to their antiplasmodial activity more precisely.

## 8. Conclusions and Future Outlook

PGM complexes have generated widespread interest in drug discovery owing to their diverse biological activities and the ability to interact with biomolecules. The emergence of resistant *P. falciparum* strains has spurred the research of these chemical entities as potential, alternative antimalarial regimens to search for candidates with unique modes of action unknown to the malaria parasite. PGM complexes show promise in this regard as demonstrated by their potent antiplasmodial effects against both chemosensitive and drug resistant strains of the *P. falciparum* parasite. Incorporation of PGM centres into bioactive ligands enhances their antiplasmodial activity, often with minimal toxicity towards mammalian cells. Remarkably, some PGM complexes do not seem to exhibit cross-resistance in in vitro biological assessment assays, a feat that can be attributed to the presence of metallic centres. While the antiplasmodial activity of most PGM complexes is well-documented in the literature, we also note that there is a great explorative scope regarding the antimalarial activity of organometallic osmium complexes as well as cyclometallated platinum compounds. Like many antimalarial compounds, PGM complexes also possess the ability to disrupt the heme detoxification pathway of the malaria parasite as a mode of action, although this property may be primarily dependent on the nature of the coordinating ligand. The affinity of PGMs for coordinating biomolecules is also illustrated by the Ru(III)Cp* complexes that interact with DNA to induce plasmodial efficacy. The suggested mode of DNA-binding involving stacking interactions with the AT-rich regions unique to plasmodial DNA could allow more specificity for targeting *P. falciparum* over the human host, paving a way for the design of PGM complexes with enhanced efficacy and lower toxicity to human cells. Application of PGM-based compounds, specifically iridium and rhodium complexes, as NAD^+^/NADH hydrogen transfer catalysts highlights the practicality of this approach not only as a novel mechanistic modality in malaria, but also as a viable avenue for lowering the effects of toxicity of antimalarial PGM metallodrugs while retaining the desired therapeutic benefit.

## Figures and Tables

**Table 1:** Antimalarial activity of Ru(II)-based thiosemicarbazone (TSC) (**40**–**43**) and quinoline (**44**–**45**) complexes against chloroquine-sensitive (CQS) NF54 and chloroquine-resistant (CQR) Dd2 *P. falciparum* strains [46,47,48,49].

Compound	IC_50_ (μM) ^a^			RI ^b^	SI ^c^
	NF54	Dd2	CHO		
**40b**	7.81 ± 0.56	– ^d^	–	–	–
**41a**	2.92 ± 0.33	4.28 ± 0.33	71.8 ± 8.11	1.47	24.6
**41b**	4.19 ± 0.12	6.66 ± 2.58	21.5 ± 0.80	1.59	5.14
**41c**	2.57 ± 0.99	2.29 ± 0.25	3.65 ± 0.630	0.89	1.42
**42a**	6.20 ± 1.00	–	–	–	–
**42b**	2.90 ± 0.90	3.8 ± 0.2	–	–	–
**43a**	16.5 ± 1.00	14.1 ± 0.90	–	–	–
**43b**	8.60 ± 0.60	6.60 ± 0.30	–	–	–
**44a**	276.8 ± 31.0	526.56 ± 70.85	–	1.90	–
**44b**	61.4 ± 10.3	>1749	–	>28.5	–
**44c**	270.2 ± 35.9	835.09 ± 190.03	–	3.09	–
**44d**	81.6 ± 7.40	228.96 ± 4.06	–	2.81	–
**44e**	71.7 ± 4.80	211.77 ± 22.68	–	2.95	–
**44f**	151.9 ± 14.1	385.61 ± 8.48	–	2.54	–
**45a**	8.27 ± 0.38	42.73 ± 12.78	–	5.17	–
**45b**	30.7 ± 2.70	42.99 ± 1.08	–	1.40	–
**45c**	4.96 ± 0.76	36.64 ± 4.33	–	7.39	–
**CQ**	5.43 ± 2.13	108.36 ± 1.10	–	19.95	–
**FQ**	42.6 ± 9.91	27.67 ± 6.46	–	0.65	–

IC_50_ (μM) ^a^ = half-maximal inhibitory concentration; activity for **44**–**45**, chloroquine (CQ), and Ferroquine (FQ) is reported in nM. RI ^b^ = resistance index, ratio of Dd2 IC_50_ to NF54 IC_50_. SI ^c^ = selectivity index, ratio of CHO IC_50_ to NF54 IC_50_. CHO = Chinese hamster ovarian. ^d^ Not determined. Data adapted from references [46,47,48,49].
